# Did tool-use evolve with enhanced physical cognitive abilities?

**DOI:** 10.1098/rstb.2012.0418

**Published:** 2013-11-19

**Authors:** I. Teschke, C. A. F. Wascher, M. F. Scriba, A. M. P. von Bayern, V. Huml, B. Siemers, S. Tebbich

**Affiliations:** 1 Max Planck Institute for Ornithology, Seewiesen, Germany; 2 Konrad Lorenz Forschungsstelle, Core facility, University of Vienna, Gruenau, Austria; 3 Department of Ecology and Evolution, University of Lausanne, Lausanne, Switzerland; 4 Department of Zoology, University of Oxford, Oxford, UK; 5 Department of Behavioural Biology, University of Vienna, Vienna, Austria

**Keywords:** tool-use, comparative cognition, corvids, Darwin's finches

## Abstract

The use and manufacture of tools have been considered to be cognitively demanding and thus a possible driving factor in the evolution of intelligence. In this study, we tested the hypothesis that enhanced physical cognitive abilities evolved in conjunction with the use of tools, by comparing the performance of naturally tool-using and non-tool-using species in a suite of physical and general learning tasks. We predicted that the habitually tool-using species, New Caledonian crows and Galápagos woodpecker finches, should outperform their non-tool-using relatives, the small tree finches and the carrion crows in a physical problem but not in general learning tasks. We only found a divergence in the predicted direction for corvids. That only one of our comparisons supports the predictions under this hypothesis might be attributable to different complexities of tool-use in the two tool-using species. A critical evaluation is offered of the conceptual and methodological problems inherent in comparative studies on tool-related cognitive abilities.

## 1. Introduction

Animal tool-use has inspired researchers of comparative cognition for decades. Much of this interest stems from the putative role of tool-use in the evolution of human intelligence. The idea is that as tools gained importance in the ecology of early hominids, selection acted upon cognitive abilities to improve their tool-using proficiency [1]. In this scenario, information-processing was honed in the context of tool manufacture and use, but gradually led to more complex and general cognitive abilities. Alternately, human tool-use may have evolved as a by-product of generalized intelligence evolved in another context (e.g. [2]). Thus, it is not surprising that much comparative research on tool-use traditionally revolved around anthropocentric questions, mainly that of whether and to what extent animal tool-use indicates continuity in the mental abilities of animals and humans.

Today, reports of tool-use in animals abound, ranging from insects to birds [3]. Animal tool-use varies tremendously in its complexity and neuronal underpinnings and thus, a fundamental revision of the original notion that tool-use is *per se* a reflection of intelligence has become necessary. Nevertheless, there are still reasons not to discard the idea of a potential link between intelligence and tool-use entirely. One is the existence of animal tool-use bearing the hallmarks of ‘sophisticated’ and potentially complex information-processing abilities, e.g. those instances involving use of multiple tools, sequential tool-use and/or complex tool modification in the wild. The rarity of sophisticated forms of animal tool-use furthermore suggests that there are constraints for its evolution, possibly cognitive abilities and/or neural substrate. Another reason is the existence of supporting correlational evidence: for birds, there is a strong relationship between tool-use and brain size [4] and tool-use and brain structure [5], and tool-use and brain size are related in primates [6]. It is therefore important to investigate the relationship between tool-use and enhanced cognition, but with a focus on identifying the kinds of cognitive tool-related adaptations to expect, and with clear predictions of where to look for them. Two approaches could be particularly useful in guiding us to answers. The first is cost–benefit analysis: tool-related cognitive adaptations are expected only where tool-use is under strong selection. Furthermore, it may help to generate hypotheses that can explain why a particular species evolved tool-use, whereas a closely related species did not. Second, once hypotheses about the relationship of tool-use and cognitive traits have been formulated, comparing cognitive performance between closely and distantly related species is essential to test them. In elaborating upon the significance of each of these approaches, we will be focusing on the context in which most tool-use occurs, namely foraging.

### (a) The cost and benefits of tool-use and their link to evolution

The main benefit of tool-use in foraging may be an increase in quantity or quality of prey that is inaccessible without tools [7]. Unfortunately, quantitative data on the nutritional benefit of tool-use are scarce [7–12]. At least for some species, it has been shown that tool-use is more costly than conventional foraging techniques in terms of time [8,9]. The costs of learning and the expenditure of cognitive resources involved in tool-use and its development are more difficult to pin down. Learning to use tools during ontogeny has been shown to be a substantial investment at least for chimpanzees (*Pan troglodytes*, [13,14]) and New Caledonian crows (NCCs; *Corvus mondeludoides*: [15–17]). Other factors and constraints that could influence the evolution of tool-use are for instance the availability of tool-materials, morphology, behavioural and cognitive traits such as sociality and motivation to play and interact with objects, neural substrate and related energetic costs [18,19]. Such limiting factors could operate to constrain or enable the evolution of tool-use at various stages, such as during its invention, its diffusion through the population and its maintenance in a population.

The interplay between costs, benefits and limiting factors can be exemplified by a comparison of the tool-using woodpecker finches (WPFs *Cactospiza pallid*a) and the small tree finch (STF; *Camarhynchus parvulus*), a sympatric non-tool-using species that is closely related to the WPF. Though they are both extractive foragers, the WPF is more specialized on extracting concealed prey from a substrate. Its straight beak is not only ideal for pecking at the substrate, but also well-suited for manipulating tools and may allow better vision at the beak tip [20]. Similarly, other characteristics related to extractive foraging such as perseverance and high levels of neophilia might have increased the likelihood for the evolution of tool-use in this species. To summarize, slight differences in ecology and morphology can significantly influence the cost–benefit ratio and the effect of constraining factors, thus indirectly influencing the evolutionary trajectory of tool-use.

### (b) The comparative approach

Traditionally, there has been a strong focus of tool-use studies on comparing tool-related complex cognition (e.g. causal understanding) between humans and various primates (reviewed in [21–24]) though a small number of studies have also investigated the tool- related cognitive abilities of non-tool-using primates [25–27].

A deviation from the focus on primates occurred with the discovery of highly sophisticated tool-use in NCCs, involving use of multiple tools to extract arthropods from various substrates and complex tool manufacture in the wild (reviewed in [28,29]). In the laboratory, they appropriately select and modify tools for a given task [30,31], display elements of planning in meta-tool tasks [32,33] and are able to develop spontaneous solutions to novel technical problems using novel materials in novel ways [34,35]. Until recently, the tool-related cognition of NCCs was considered unique among corvids. However in 2009, rooks (*Corvus frugilegus*), a non-tool-using corvid species, were found to perform similarly to NCCs in several cognitive tool-related tasks [36]. This suggests that the special cognitive abilities of NCCs may have evolved in another context preceding the evolution of tool-use [2].

The findings of Emery & Bird [36] remind us of the importance of a rigorous comparative programme to determine whether there is a general pattern of association between an adaptive cognitive specialization and tool-use. This should not only entail comparisons between distantly related tool-users, but also between closely related tool-users and non-tool-users. Thereby, repeated appearance of the cognitive trait in question only in tool-users in phylogenetically independent species pairs would be strong evidence for a cognitive adaptation [37].

## 2. Our study: test of tool-related physical cognition in corvids

Over the past 5 years, we have applied the comparative method, as described earlier, to two species pairs to investigate the question of whether the use of tools evolved in conjunction with enhanced and specialized physical cognitive abilities. Previously, we compared general learning and physical cognitive abilities in the WPF and a closely related non-tool-using species, the STF. WPFs habitually use twigs or cactus spines to extract arthropods from crevices and show simple modification of these tools such tool shortening or breaking of side twigs [8].

We predicted that if tool-use evolved with enhanced specialized cognitive abilities, WPFs should outperform STFs in physical tasks but not necessarily in the general learning tasks. Contrary to expectation, the STFs performed similarly or better than WPFs in one of the general learning tasks and most of the physical tasks [38,39]. Thus, the study yielded no evidence that WPFs have enhanced physical cognition.

The aim of this study was to provide highly comparable data from a new tool-using/non-tool-using species pair from a distantly related group using the methods which had been applied in the preceding Darwin's finch study. This allows a more complete assessment of the hypothesis that tool-use might generally be associated with adaptive cognitive specializations in the physical domain. Using the paradigms previously established with Darwin's finches, we tested physical cognition and general learning abilities in tool-using NCCs and a non-tool-using species, the carrion crow (*Corvus corone*). To our knowledge, ours is the only study to rigorously apply the comparative method to interspecific comparisons of closely related tool-using/non-tool-using species pairs in two distantly related groups and using identical learning paradigms to test all the species involved.

### (a) Methods

#### (i) Study locations, subjects and housing

All NCCs and one hooded crow (*Corvus cornix*) were tested at the Avian Cognition Research Station (ACRS) hosted by the Max Planck Institute for Ornithology (MPIO), Seewiesen, Germany from March to November 2011. All carrion and the remaining hooded crows were tested at the Konrad Lorenz Research Station (KLF) in Grünau, Austria from June to August 2011 and at the MPIO in June 2009 and November 2009–May 2010. The test for neophilia (see below) was conducted in 2012 at the ACRS and KLF, and the NCCs were not the same as the ones tested in the other tasks (see the electronic supplementary material, table S1).

The data from two hooded crows were included in the carrion crow dataset as these closely related species have been only recently split into two separate species and share similar ecology and morphology [40]. Although the hooded crows are treated as carrion crows for the statistical analysis, these individuals have been made distinguishable from the carrion crows in the graphs. Furthermore, some WPFs do not use tools (detailed explanation in the methods of the electronic supplementary material, see also [38]). The WPF dataset is therefore comprised tool-using and non-tool-using individuals, and this distinction is taken into account in the analysis. A summary clarifying the order of experiments, the participation of each bird in each experiment as well as rearing history is given in the methods section and the electronic supplementary material, table S1. Further details concerning rearing experience and housing are also in the methods section of the electronic supplementary material.

#### (ii) Experiment 1: the cane task

**Figure 1.**
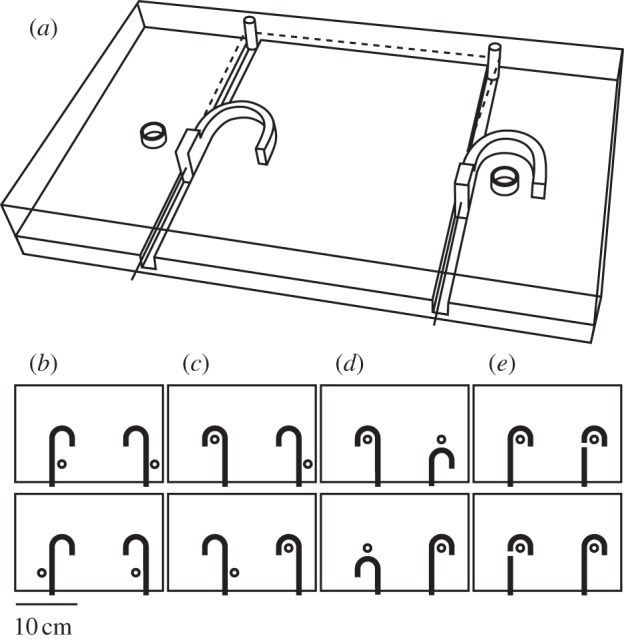
(*a*) The cane task apparatus with canes and rewards arranged as in the initial test condition. Panels (*b*)–(*e*) transfer tasks 1–4. Adapted from [38,39].

This experiment involved food retrieval contingent on making a choice between two canes, only one of which could retrieve the reward. In each condition, one food reward was inside the hooked portion of the cane and one outside of it (with the exception of transfer task 4 in which both rewards were inside the hooked portion of both canes; figure 1). Eight NCCs, five carrion crows, two hooded crows and 12 WPFs participated in this experiment.

*Transfer tasks*. Those subjects that solved the initial version of the task by reaching the set learning criterion (see below) within a maximum of 180 trials were then tested in four further variations of the initial task (transfer tasks, figure 1*b–e*), where they received a maximum of 20 trials in each.

#### (iii) Experiment 2: the reversal task

The apparatus consisted of two feeders covered with coloured lids (orange and blue) that were mounted 30 cm apart on a grey Perspex base (50 × 50 cm). In each trial, a reward was placed in one of the feeders, the lids were placed on the feeders and birds were then allowed to remove one of the two lids. A transparent Perspex divider prevented the birds from removing the lid of both feeders. The experiment consisted of two phases: an initial ‘acquisition phase’ and a ‘reversal phase’. In the acquisition phase, only one colour was the rewarded (S+) stimulus. Half of the birds started with orange as S+ and the other half with blue as S+. Once a subject met criterion (see general experimental procedure), the colour-reward contingency was reversed in the reversal phase. Subjects were given a maximum of 140 trials in each phase. Eight NCCs, seven carrion crows, one hooded crow and 16 WPFs were tested in this experiment.

#### (iv) Experiment 3: the novel box-opening task

The apparatus was a box made of opaque, white Perspex with a transparent lid (box: width = 15 × depth = 12 × height = 12 cm; lid overlaps edge at front by 3 cm). The lid was hinged to the back edge of the box and overlapped the front edge of the box. The box could be opened by pushing the protruding lip of the lid upwards. Before testing, birds were habituated to the box by feeding from it once while it was open. Subjects were given six sessions of 25 min, receiving up to three sessions per day. A bird was successful and testing was ended when it opened the box and ate the reward. If a bird did not make physical contact with the box during a session, it was re-habituated to the box as described above and the session was repeated. A bird was given up to two extra sessions upon failing to make contact with the box in any one session. Eight NCCs, seven carrion crows and two hooded crows were tested in this study. Additionally, we compare the NCC data to that of 18 WPFs which were tested for a previous study [38]. The ‘total length of testing’ (s) and ‘success’ (opening box and gaining access to the food reward) were the measurements taken for comparison.

#### (v) General experimental procedure

Experiments were conducted in the home aviaries of the birds, and food was removed from their aviaries approximately 2 h before testing. The apparatus was baited out of sight of the subject, and for each trial, placed onto the experimental table or experimental area within the home aviary. The experimenter then left and observed the trial via camcorder.

Experiments 1–2 were two-choice learning experiments involving the same basic procedure. These tasks were conducted in blocks of 10 trials. In each trial, the subject was given 5 min to choose between two options for which the correct side was pseudorandomized and counterbalanced right and left. Some slight modifications to the original testing conditions and testing regime with WPFs were made with the corvids (see the electronic supplementary material, methods).

*Learning criterion.* To meet the success criterion, a bird had to make 15 or more correct choices within two consecutive blocks of 10 trials. Specifically, the number of correct responses in one of the two blocks had to be at least seven consecutively correct and in the other at least eight or in one block all 10 correct. This criterion was derived using a Monte Carlo simulation (details in [41]). Some subjects developed a positional bias, which we defined as six consecutive choices of one side, probably owing to intermittent reinforcement. When this happened, we applied a side bias correction procedure (‘correction trials’, see the electronic supplementary material, methods for details).

#### (vi) Statistical analysis

Fisher's exact test was used to test for species differences in the proportion of individuals to meet the success criterion. We also compared learning speed and success probability between NCCs and carrion crows/hooded crows and between NCCs and WPFs in the initial phases of the cane task and for both phases of the reversal task using generalized linear mixed models, specifying logistic regression with binomial errors (GLMM, [42]). For each species comparison, a separate model was constructed for the initial phase of the cane task and for both phases of the reversal task. Prior to fitting the model, we *z*-transformed trial number to a mean of 0 and a standard deviation of 1, and side bias correction trials were removed from all datasets. Full details of the modelling process and the full model results can be found in the methods and results sections, electronic supplementary material, tables S2–S4.

Each statistical comparison between NCCs and WPFs was conducted once only with tool-using WPFs and once with all WPFs pooled (details in the electronic supplementary material, methods). Since the conclusions always remained the same regardless of which WPF grouping was used, we report only results for the comparison of NCCs with pooled WPFs.

All statistics were conducted with R v. 2.15.1 [43].

#### (vii) Comparison of novelty reactions and motivation between corvid species

Novelty reactions between carrion crows and NCCs were assessed in two different ways.

First, we presented individuals with a novel object (green dog-toy ball, electronic supplementary material, figure S1) and measured latency to first contact. Birds received one 10 min session per day during which they were separated from other birds. If they did not approach the object within a session, the novel object was removed and the individual received another session the next day. The test ended after the session in which the birds first touched the object or after 13 sessions, if individuals did not approach before. Each bird received a value calculated by adding the total session time until it touched the object for the first time. Birds that did not touch the ball within 13 sessions received a ceiling value which was calculated by adding one to the highest latency to touch the ball among the other crows. The Mann–Whitney *U*-test was used to compare ball touch latencies between the species.

Second, we measured the latency to approach the cane apparatus and also to touch the cane averaged over the first 50 trials for each individual (details in the electronic supplementary material). While strictly speaking, these do not provide measures of novelty reaction, they do however provide a way of comparing the motivation with which these animals interacted with the cane apparatus. We deemed this important as carrion crows are known to be neophobic, when approaching novel food [44] and differences in motivation could potentially explain differences in cane task performance. These measures were scored from videos by M.F.S. In trials where the birds did not approach the apparatus at all in 5 min, birds were given a ceiling value, which was derived by adding 1 to the maximum time in each category. The Mann–Whitney *U*-test was used to compare apparatus approach and cane touch latencies between the species.

### (b) Results

#### (i) Experiment 1: the cane task

**Figure 2.**
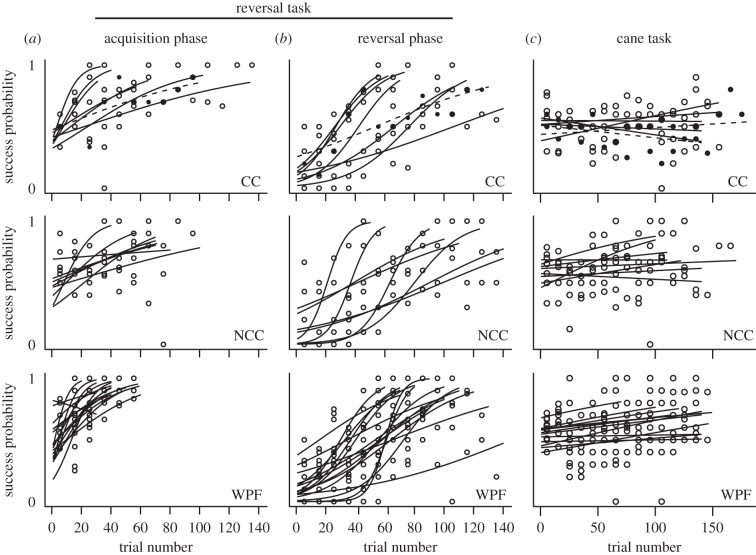
Logistic regression models of success probability across trial number for two-choice learning tasks. (*a*) Acquisition phase and (*b*) reversal phase in the reversal task, (*c*) the cane task. Each row refers to one of the three test groups (CC, carrion crows; NCC, New Caledonian crows; WPF, woodpecker finches. Points refer to the proportion of correct trials per block of 10 trials for each individual. Data and models from the hooded crows, a subgroup of the carrion crows, are denoted by dashed lines and solid black points. Graphs from WPFs were adapted from [38].

Neither the five carrion crows nor the two hooded crows were able to solve the initial task, while five of the eight NCCs and eight of the 12 WPFs met the learning criterion in this phase. The proportion of NCCs and WPFs to reach criterion in the initial task did not differ significantly (Fisher's exact test: *p* = 1). However, proportionally more NCCs than carrion crows solved the initial task (Fisher's exact test, *p* = 0.026). The disparity in learning performance in the initial task between NCCs and carrion crows was further reflected in the significant difference in the speed with which they improved in this task (corvid model: species × trial: χ^2^_1_ = 4.13, *p* = 0.042; figure 2).

NCCs and WPFs did not differ significantly in their learning speed of the initial task (tool-user model: species × trial: χ^2^_1_ = 0.001, *p* = 0.973; figure 2) nor did they differ in their overall success probability (tool-user model: species: χ^2^_1_ = 2.21, *p* = 0.137; figure 2). Nevertheless, they generally learned over the course of trials (tool-user model: trial: *z* = 4.98, *p* < 0.001; figure 2).

*Transfer performance.* The five NCCs and eight WPFs that successfully solved the initial task were subsequently tested in four transfer tasks. Only one WPF and no NCCs met criterion in the first transfer task. Two NCCs and one tool-using WPF met criterion in the second transfer task. As we found previously, performance was best in the third transfer task where three NCCs and six WPFs met criterion. No individuals solved the fourth transfer task.

To further assess transfer differences between NCCs and WPFs, we pooled data from the first two transfer tasks for each bird and calculated the proportion of trials in which birds made a correct choice to the total trials received in the first two sessions. We used only data from the first two sessions of each transfer, because the sample size of NCCs declined to four after the second transfer task since one subject fell ill. There was no significant difference in transfer performance between NCCs (median proportional correct = 1.58, range = 1.21–1.74) and WPFs (median = 1.79, range = 1.53–2.22) according to this measure (Mann–Whitney *U*-test: *N*_NCC_ = 5, *N*_WPF_ = 8; *U* = 9; *p* = 0.124).

#### (ii) Experiment 2: the reversal task

All individuals met the learning criterion during the acquisition phase and also in the reversal phase, with the exception of one carrion crow, two NCCs and one WPF in the reversal phase. NCCs and carrion crows did not differ in their speed of learning either in the acquisition phase (acquisition phase model: group × trial number: χ^2^_1_ = 0.02, *p* = 0.882; figure 2) or in the reversal phase (reversal phase model: species × trial number: χ^2^_1_ = 0.14, *p* = 0.711; figure 2) although individuals clearly improved in both phases of the task (acquisition phase-corvid model: trial number: *z* = 6.52, *p* < 0.0001; reversal phase model: trial number: *z* = 6.92, *p* < 0.0001; figure 2). Overall success probability also did not differ between these species in the acquisition phase (species: χ^2^_1_ = 0.17, *p* = 0.682; figure 2) and in the reversal phase (species: χ^2^_1_ = 0.76, *p* = 0.383; figure 2).

In the acquisition phase, WPFs were significantly faster in their learning speed than NCCs (acquisition phase model: species × trial number: χ^2^_1_ = 10.08, *p* = 0.001; figure 2) but not in the reversal phase (reversal phase model: species × trial number: χ^2^_1_ = 0.001, *p* = 0.974; figure 2). There was no species difference in overall success probability in the reversal phase (reversal phase model: species: χ^2^_1_ = 0.0005, *p* = 0.982; figure 2) though individuals clearly improved over trials in this phase (reversal phase model: *z* = 8.53, *p* < 0.001; figure 2).

#### (iii) Experiment 3: the novel box-opening task

Five NCCs, eight WPFs and six carrion crows successfully solved the task within six sessions. There was no significant difference in proportional success between carrion crows and NCCs (Fischer's exact test, *p* = 1), nor was there a significant difference in proportional success between NCCs and WPFs (Fisher's exact test, *p* = 0.673).

*Comparison latency to success.* When the length (in seconds) of all sessions was summed for each individual and species comparisons conducted, we found no significant differences either between NCCs (median = 6269 s, range = 82–9000) and carrion crows (median = 1740 s, range = 34–9000; Mann–Whitney *U*-test: *N*_CC_ = 9, *N*_NCC_ = 8, *U* = 30.5, *p* = 0.623) or between WPFs (median = 9000 s, range = 50–9000) and NCCs (Mann–Whitney *U*-test: *N*_WPF_ = 18, *N*_NCC_ = 8, *U* = 55, *p* = 0.327).

#### (iv) Comparison of novelty reactions and motivation between corvid species

There was no evidence of a significant species difference either in the median mean times to approach the cane apparatus (carrion crows: median approach time = 6.16 s, range = 4.68–48.48; NCCs: median = 6.75 s, range = 2.12 –21.98; Mann–Whitney *U*-test: *N*_CC_ = 7, *N*_NCC_ = 8, *U* = 18, *p* = 0.281) or in the median mean times to touch the cane (carrion crows: median cane touch time = 8.92 s, range = 6.74–61.20; NCCs: median = 8.79 s, range = 4.42 –25.78; Mann–Whitney *U*-test: *N*_CC_ = 7, *N*_NCC_ = 8, *U* = 19, *p* = 0.336) during the first 50 trials.

There was no significant difference between NCCs (median latency = 7475 s, range = 328–7475) and carrion crows (median latency = 3152 s, range = 4–7474) in the latency with which they touched the ball (Mann–Whitney *U*-test: *N*_CC_ = 7, *N*_NCC_ = 8, *U* = 12, *p* = 0.068).

### (c) Discussion and main conclusions from our study

Our previous study [38] did not confirm the presence of enhanced physical cognitive abilities in the WPF, when compared with a closely related non-tool-using species. However, in line with the prediction that tool-use evolved with enhanced physical cognitive abilities, this study found that NCCs outperformed carrion crows in the cane task, but not in two general learning tasks. This difference in corvids but not Darwin's finches matches the expectation that sophisticated cognitive tool-related adaptations should be found in species with sophisticated tool-use, as NCCs substantially exceed WPFs in tool diversity and the complexity of tool manufacture (reviewed in [28]).

The difference in NCC and carrion crow performance cannot be explained by different levels of neophobia or motivation, because we found no significant difference in either of our two measures between the two species, but it might be attributable to the unaccustomed cognitive load that the manipulation of a tool imposed on the non-tool-using species [45].

However, there are several reasons to treat the results with caution. One is that the comparison groups of corvids did not share identical rearing conditions: most of the NCCs were wild-caught, whereas all of the carrion crows were hand-raised. Although this could have had a substantial effect on the results, it is difficult to predict exactly in which direction. Another reason is that none of the carrion crows reached the learning criterion in the cane task and, thus, the performance of this large-brained corvid was worse than that of the STFs. This is surprising, especially given the excellent performance of other non-tool-using corvids, such as rooks in physical tasks [36,46].

Also, the comparison of large-brained corvids and distantly related WPFs did not expose a significant difference in physical task performance between these tool-using species either in the initial cane task or in the cane transfer task performance. The lack of a difference in the cane transfer tasks is very surprising given the performance of NCCs in other tasks that indicate at least some sensitivity to physical rules and transfer to similar problems [35,47,48]. One way of understanding these results is that NCCs may simply use simple perceptional cues in some situations but not in others [23,49]. Also, perhaps the cane task poses a problem that is irrelevant to these species’ natural tool-use, and thus imposes an unanticipated measure of difficulty on both species. Finally, it may be that the performance of NCCs, who usually engage in sophisticated tool-use, may somehow be impaired when forced to engage in artificial tasks involving choices between two options [50] or involving restricted object manipulation as necessitated by the cane task. For future studies, we suggest inclusion of two groups of tool-users: one which uses freely manipulable tools and one which uses the pre-inserted tools. We further recommend, where possible, the avoidance of binary choice tasks.

Our data on the physical cognitive adaptive specialization hypothesis are subject to some of the criticisms shared by many studies of comparative cognition. For one thing, cognitive characteristics other than the one of interest could have been taxed by the tasks, leading to a pattern that does not fit our prediction. We also may have missed the crucial subcategory of physical cognition that differs between the species, having only presented one test of physical cognition. Finally, the lack of identical rearing conditions between groups is a common problem with long-lived and unusual species that require significant funds to raise and maintain. These problems are linked to some of the more universal challenges confronting researchers in comparative cognition. In the following, we address some potential solutions for these challenges and touch upon our suggestions for future research on tool-related cognition.

## 3. Outlook

### (a) Troubleshooting the physical cognitive adaptive specialization hypothesis: getting ahead of ourselves?

The ecological approach in understanding adaptive behaviour and underlying cognitive mechanisms is an iterative process of investigation, synthesizing hypotheses and predictions both from the functional and mechanistic perspective. A classic example of this process has been described in detail by Smulders *et al*. [51] for food caching. Differences in brain morphology between caching and non-caching species and seasonally varying brain morphology in several food-caching species from parids to corvids have been central findings fuelling the cognitive adaptive specialization hypothesis for food storers (reviewed in [52]). In the food-caching paradigms, ecological knowledge not only helped to point to memory as a likely solution to the problem which food hoarders face in the wild, but later it helped to refine the hypothesis to fit various ecological circumstances of food hoarding animals. More specifically, the realization that only a subgroup of food hoarders need to remember cache locations for a long period lead to the proposal that only these species should have a duration of spatial memory longer than that of other species. Thus, the spatial memory hypothesis for food hoarding comes from knowledge of the function of food hoarding in animals and is informed by the details of ecology.

In the case of tool-use, there has been a disproportionate focus on human-like complex cognitive mechanisms like causal understanding that may be involved in animal tool-use. However, gradual improvements through associative processes (i.e. associative learning of spatial relationships, attention towards relevant cues) could improve efficiency of flexible and context-specific tool-use just as well as complex mechanisms and thus should be included in predictions. Also, recent data suggest that the underlying cognitive mechanisms of even supposedly sophisticated tool-users such as chimpanzees and NCCs are a mixture of complex and simple mechanisms and those in between [23]. We suggest that future studies put less weight on distinguishing between complex and simple cognition in tool-use and that they are careful to root predictions about tool-related cognitive mechanisms in detailed examination of the tool problems faced by animals in their natural context.

### (b) Roadmap for future comparative studies of tool-use and cognition

As we see it, there are currently two main approaches to take in advancing our understanding of the cognitive mechanisms underlying tool-related cognition. One is to refine hypotheses, whereas the other is to refine methodology.

#### (i) Using knowledge of tool-use in a natural context to refine hypotheses

NCCs exemplify how knowledge of ecology and natural history has been useful in suggesting areas of cognition for experimental investigation. For example, geographical variation in NCC tools has been touted as suggestive of cumulative cultural evolution [53]. Along with other field studies that have demonstrated the existence of ample opportunities for social transmission of information in this species, this suggests that it may be promising to look at social learning in NCCs [53]. Furthermore, the observation that NCCs use barbed pandanus tools in the appropriate orientation in the wild (in order to be functional the tool must be held such that the hooks on the barbed edge face backwards from the tool tip) lead researchers to question the cognition involved in this behaviour [54]. One explanation put forth was that NCCs might have a human-like underlying understanding of the physical properties of hooks, leading to an understanding of the functional features of the tool. Another is that the behaviour might also be the result of simple associative mechanisms—possibly trial and error learning. Subsequent experimental investigation with wild-living crows or those held briefly in captivity showed limited attendance of the crows to the functional orientation of the barbs and also that when tools are dropped and/or picked up in the non-functional orientation, repositioning arises from simple associative strategies. In conclusion, the authors state their view that correct functional tool orientation in the wild may simply be the outcome of applying the correct procedural rules of pandanus tool manufacture ([54], but see also [55]). This is a laudable example of an observation from the wild that was rigorously tested using semi-wild experiments that were devised to differentiate between the so-called high-level and low-level underlying cognitive abilities. Its results clearly illustrate that what seems complex is not necessarily so. Other observational field studies on bearded capuchin monkeys (*Sapajus libidinosus*) and chimpanzees inspired the hypothesis that enduring artefacts play an important role for some nonhuman species in learning to use tools by supporting persistent practice [56]. To summarize, detailed knowledge of the behaviour from wild populations will help to identify the type of the problems that animals are faced with in nature and thus generate more accurate hypotheses concerning the underlying mechanisms of their behaviour. Thereby, it is essential to adhere to the principle of parsimony and presume the lowest denominator of cognitive mechanisms required for solving the problem. Improved knowledge of ecology and tool-use in a natural context will help to formulate more precise and appropriate predictions concerning which specific cognitive mechanisms might be linked to tool-use.

#### (ii) Improving methods

Not only do we need more background information on species’ natural behaviour to inform predictions, but we could also benefit greatly from improvements in methodology. Much would be gained if more studies adopted a rigorous comparative approach with interspecific comparison between at least one tool-using and one non-tool-using species, using the same or at least a comparable methodology. Including more non-tool-using species in the comparison will furthermore ensure robustness of an observed pattern. Another huge challenge of comparative cognition is that we often are unaware of the full suite of cognitive mechanisms influencing performance in a given task. Consequently, we are often not clear about the questions posed, an issue which is compounded in multi-species comparisons. Ideally, task designs should account for the different species ‘non-focal’ cognitive traits, that is, those traits which are not at the focus of an experiment, but can nevertheless have a strong, sometimes central influence on task performance, such as attention, motivation and temperament but also species differences in perception [57,58]. A good example of this approach is the study of Chappell *et al.* [59] that presents a detailed analysis of the relationship between perseverance and impulsivity on innovation in tool-use problems with human children. One solution is to develop task batteries that include tests of such general psychological variables in addition to those specifically probing only the cognitive trait(s) of focal interest [58,60]. Here, it is important to ‘find the right items to test’ by rooting predictions in species ecology and observation and the tasks must be revised as necessary. There is one major conundrum inherent in the task battery approach for multiple species (and also more generally in comparative cognition): in order to yield comparable data, tasks must be standardized, yet some species-specific task modifications are a ‘necessary evil’ to increase ecological validity and the likelihood that each species is being asked the same question. The crux of the problem is that we often do not know in advance what the cognitive, motor and perceptual disparities between species are and how they will influence task performance. Danger lies in succumbing to the temptation of ‘quick and dirty’ experiments that simply apply pre-existing paradigms to new species without sufficient thought devoted to tailoring the problem to the species at hand [61]. Intimate knowledge of a species through careful observation both in the field and in the laboratory is the best help in designing comparable studies and ecologically valid paradigms. In combination with an improved understanding of animal's overall psychological profiles (i.e. motivation, temperament, motor and perceptual abilities, etc.), a useful framework inspired by Artificial Intelligence could help to decompose demands of cognitive tasks into their component parts [62]. This would help clarify the nature of the problem, taking into account the information available to the animals and possible problem solutions.

One further issue we faced in this study is the interpretation of failure. Should we accept the null hypothesis that carrion crows simply do not have enhanced physical cognition? Our comparative results suggest that this may not be justified and that further testing is needed to understand these results. Seed *et al*. [63] recently reviewed this topic in detail and suggest that understanding failures can be achieved, for example, by deconstructing a task and lowering task demands while asking the same question and by testing the species in several different analogous tasks [64]. Another way to understand failures is to look at whether the difference between individuals that pass and fail can be correlated with variation in another psychological trait [63]. These approaches might be fruitful in future attempts to understand our results.

To conclude, in the field of comparative cognition, considerable progress has been made by adopting an integrative approach in understanding animal cognition [65]. The process of the ecological approach is nonlinear, oscillating between predictions generated from functional and mechanistic knowledge of a behaviour. Thus, an understanding of the relationships between cognition and behaviour requires consideration of both ultimate and proximate standpoints. The best approach to choose at a given time is determined by common sense. However, the ecological context of behaviour must always be kept in mind, especially during laboratory studies of mechanism.

## Notes

Permission to conduct experiments with the woodpecker finches was granted by the Galápagos National Park and the Charles Darwin research station (Project PC-16-07, permit no. PR.PT.P004.R02). For the experiments with the crow species at MPIO, permission was granted by the government of Upper Bavaria in Germany (Az. 55.2-1-54-2531-87-08). The work with the carrion crows at KLF complied with guidelines provided by the Austrian Federal Act on the Protection of Animals (Animal Protection Act -§ 24 Abs. 1 Z 1 and 2; § 25 Abs. 3 – TSchG, BGBl. I Nr. 118/2004 Art. 2). Furthermore, as our experiments are purely appetitive, strictly non-invasive and based exclusively on behavioural tests, they are classified as non-animal experiments in accordance with the Austrian Animal Experiments Act (§ 2. Federal Law Gazette No. 501/1989) and no licence was required. Experiments were conducted by an experienced animal caretaker, ensuring no animals were harmed by the experimental procedure. All individuals participated and entered the experimental compartments voluntarily. Focal individuals were rescued from the wild when taken out of the nest at a young age by unfavourable weather conditions. Individuals were held in captivity at the KLF (licence AT00009917) before and after completion of the present study.
